# Quality of Life and Psychiatric Comorbidities among Subjects Practicing Artificial Skin Depigmentation in 2020 in the City of Cotonou (Benin)

**DOI:** 10.1155/2024/8589329

**Published:** 2024-04-23

**Authors:** B. Degboe, M. M. D Baloubi, N. Ntouala Noukayaba, F. G. Akpadjan, H. Adegbidi, F. Atadokpede

**Affiliations:** ^1^Service de Dermatologie-Vénérologie, Centre National Hospitalier et Universitaire Hubert Koutoukou Maga de Cotonou, Faculté des Sciences de la Santé-Université d'Abomey-Calavi (Cotonou-Bénin), Campus Universitaire Champ de Foire 01 BP. 18, Cotonou, Benin; ^2^Unité d'Enseignement et de Recherche en Pharmacie, Faculté des Sciences de la Santé-Université d'Abomey-Calavi (Cotonou-Bénin), Campus Universitaire Champ de Foire 01 BP. 18, Cotonou, Benin

## Abstract

**Introduction:**

The purpose of this study was to study the quality of life and psychiatric comorbidities of subjects practicing voluntary skin depigmentation in the city of Cotonou.

**Methods:**

A cross-sectional, prospective, and analytical study, based on a three-stage probabilistic sampling method, included from June to October 2020, consenting subjects over 15 years of age, practicing artificial skin depigmentation, and residing for at least one year in Cotonou. The Dermatology Life Quality Index, Rosenberg, and Hospital Anxiety and Depression scales allowed us to evaluate the quality of life and self-esteem, and identify anxiety and depression, respectively. A *p* value <0.05 indicated a significant result.

**Results:**

We included 330 subjects. The mean age was 33.6 ± 11.6 years and the sex ratio was 0.4. Impaired quality of life was observed in 93.7% of subjects. Anxiety was diagnosed in 11.2% and depression in 5.8% of them. Self-esteem was low or very low in 24.2%. The degree of quality of life and the alteration of self-esteem, and the frequency of anxiety and depression were proportional to the number of skin lesions, the lightening products used, and the monthly cost of the products.

**Conclusion:**

The use of several lightening products exposes patients to numerous skin lesions, which are a source of impaired quality of life and whose persistence leads to psychiatric comorbidities.

## 1. Introduction

Artificial depigmentation is a very common practice in black-skinned populations, involving the lightening of the natural color of the skin, at the risk of sometimes dramatic complications [1–6]. These complications are dermatological, systemic, and psychiatric [3, 7–9]. Psychiatric complications are characterized by addiction, altered quality of life, low self-esteem, anxiety, depression, and suicidal ideation [4, 10–13]. However, little is known in the literature about the quality of life and psychiatric comorbidities associated with dermatological complications in subjects practicing depigmentation. This led to the present study. The aim of this study was to investigate the quality of life and psychiatric comorbidities in subjects practicing voluntary skin depigmentation in the city of Cotonou.

## 2. Methods

This study took place in each of the 13 districts of the city of Cotonou. It was a prospective cross-sectional study with analytical objectives, conducted from June to October 2020. The sample size was calculated using the Schwartz formula. We used a probabilistic three-stage sampling method. A district per arrondissement was selected at random. In each district, we selected a single neighborhood using simple random sampling. Once in the selected neighborhood, we employed the bottle method. The subjects for the survey were chosen based on convenience, meaning we selected them according to the ease of access, while still adhering to the inclusion criteria until the desired sample size was achieved. Subjects aged 15 and over, practicing voluntary skin depigmentation, residing for at least one year in the neighborhood where the survey was carried out, and having given their free and informed consent were included in the study. A structured interview conducted in French and based on the pre-established questionnaire, was used to collect information on various variables. In cases of language barriers, the interviewers translated the questionnaire to the respondents without influencing their answers. First, we collected sociodemographic data; second, we listed the cosmetic products used and the dermatological complications presented by the subjects surveyed; and third, we assessed the quality of life and identified any psychiatric comorbidities using specific scales.

The Dermatology Life Quality Index (DLQI) is the standard tool for measuring the quality of life in dermatology. It consists of ten questions regarding the impact of skin problems on the subject's life over the past week. The questions cover symptoms, feelings, daily activities, leisure, work or school, personal relationships, and treatment. Patients answer each question by ticking one of the following boxes: “not at all,” “a little,” “a lot,” and “a great deal.” The score for each question is between 0 and 3, for a total score of 30 [14].

The Hospital Anxiety and Depression Scale (HADS) [15, 16] is a 14-item self-questionnaire that assesses anxiety and depression over the past week. Seven items, originally present in the Clinical Anxiety Scale, concern psychological anxiety. The depression subscale items assess dysphoria (1 item), slowing down (1 item), and anhedonia (5 items). Each item is scored from 0 to 3. The threshold score of 10 is proposed by many authors for each of the subscales.

The Rosenberg scale is used to assess self-esteem. The questionnaire consists of 10 items, 5 of which assess positive self-esteem and 5 negative self-esteem. The response varies according to a four-point Likert-type scale ranging from “totally disagree” (1) to “totally agree” (4). The score is obtained by adding the scores for questions 1, 2, 4, 6, and 7. For questions 3, 5, 8, 9, and 10, the scoring is reversed. The score is between 10 and 40 [17]. Data were entered using EPI Data version 3.1 and exported to SPSS and Excel 2013 for processing and analysis. The chi-square test with *p* value was performed at a significance level below 0.05. Pearson's coefficient was used to determine the correlation between the dependent variables in terms of scores and the study's quantitative independent variables, as well as among the dependent variables themselves.

## 3. Results

A total of 330 subjects were surveyed. Their average age was 33.6 ± 11.6 years, with a predominance of the 25–30 age group. Our study population was predominantly female, with 232 women (70.3%) versus 29.7% men. Among subjects practicing artificial depigmentation, bachelors (42.7%) were the leaders, followed by married (25.7%) and civil partners (19.4%). The most common levels of education were secondary (40.3%) and higher (38.2%). Most were civil servants (22.7%), shopkeepers (18.8%), and craftsmen (15.8%).

The respondents used soap (97.9%), body lotion (92.5%), lotion (39.7%), and other lightening products (31.5%). Most, 77.3%, used 2 or 3 products at a time. The average monthly expenditure on products was 11120 ± 13670 FCFA, with extremes of 1500 and 32000 FCFA.

**Figure 1:**
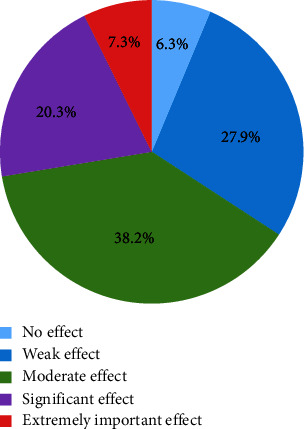
Distribution of 330 subjects undergoing artificial skin depigmentation in the city of Cotonou in 2020 according to the quality of life score.

The various skin complications observed in our study were dominated by dyschromia (75.8%), stretch marks (57%), superficial mycoses (46.1%), and acne (41.5%). Of the 314 subjects with skin complications, more than half (54.5%) had more than 2 skin complications. The mean quality of life score of the subjects surveyed was 9.3 ± 3.3, with extremes of 0 and 28. The quality of life was altered in 93.7% of the subjects (Figure 1).

Skin lesions due to the consequences of voluntary skin depigmentation significantly impacted the quality of life of young and female subjects, who were mostly single and had a secondary or higher level of education (Table 1). There was an increasing trend between the impairment of quality of life and the number of lightening products used, the number of skin complications, and the monthly cost allocated to depigmenting products (Table 1).

The mean anxiety score of the subjects surveyed was 8.3 ± 2.1, and 11.2% of them were anxious. Most of the anxiety subjects were over 50 years old, female, single or married, with secondary or higher education, and were employees or shopkeepers (Table 2). As the number of products used increased, so did the level of anxiety. The same was true for the number of skin lesions and the monthly cost allocated to these products (Table 2).

The mean depression score was 7.4 ± 2.8 and 5.8% were depressed. Most of the subjects with depression were younger (20–40 years), single, married, or divorced, with secondary or higher education, and were wage earners or shopkeepers (Table 3). Subjects who used more than 3 products at a time, had more than two skin lesions, and spent more than 10,000 FCFA on the monthly purchase of these products were more depressed (Table 3).

The mean self-esteem score for the subjects surveyed was 32.1 ± 3.1, and 24.2% of the subjects had low to very low self-esteem. Most subjects with low to very low self-esteem were single, shopkeepers, and had a high school education (Table 4). The degree of self-esteem was inversely proportional to the number of skin lesions, the number of lightening products used, and the monthly cost allocated to these products (Table 4).

## 4. Discussion

The 20–40 age group remains the most concerned in all sub-Saharan African studies. The prevalence of voluntary depigmentation at this age can be explained by the more active search for a partner or the maintenance of a relationship for the purpose of seduction [1–7]. The trend of females observed in our study has also been reported in most previous studies. Regarding the marital status of our respondents, the desire to be more beautiful, the main goal sought by female practitioners, would be more present among these single women, still looking for a partner, or among married women where there is rivalry between the wives of the same husband in a polygamous context [1–4, 11, 18]. As reported by some authors, women with a high level of education practice voluntary depigmentation more than artisans and housewives. This is because they are more easily influenced by magazines, the media, and the Internet, thanks to their reading skills [2, 19–21]. When comparing our results with the DLQI averages found in other studies, we note that the average score for quality of life impairment in our subjects was higher than that found in most other diseases [22–28]. In fact, there is a very close link between beauty and well-being. Artificial depigmentation, regarded as the indispensable means of achieving the beauty sought after by practitioners, is more likely to lead to a deterioration in the quality of life than other dermatoses, when it leads to the opposite and disastrous results [4, 10–13]. We note that subjects who used more cosmetic products had a greater deterioration in quality of life. In fact, skin lesions secondary to artificial depigmentation are an addictive phenomenon in practitioners. They often hope to finally obtain a positive or better result. However, the accumulation of depigmenting products leads to a worsening of preexisting lesions or the appearance of new ones, which contributes to a further deterioration in their quality of life.

Only dyschromia, acne, and superficial mycoses were significantly associated with poor quality of life. These complications were found in several other studies at varying frequencies [1–9, 18–21]. Dyschromia and acne are highly visible lesions, often located in exposed areas of the body and therefore more difficult to conceal. This leads to greater aesthetic prejudice in subjects in search of seduction and relational difficulties, in short, an alteration in the quality of life, leading to avoidance or even isolation.

Anxiety affected the older subjects in our population, while depression affected the younger ones. Younger people encourage themselves to continue their practice in the hope of better results. This leads them to use products with a more “powerful” lightening effect. They end up feeling depressed about the skin disasters caused by these products. Older subjects, on the other hand, start to worry about their skin lesions, which are generally no longer recent. Instead, they fear a malignant transformation of the lesions or a systemic disease in which the skin lesions are just the tip of the iceberg. This situation leads to constant fear, hence the high prevalence of anxiety at this age. However, it should be noted that 10.5% of subjects with definite symptoms of depression had no skin lesions whatsoever. In fact, some of the participants in our study used skin-lightening cosmetics but saw no effect on their skin. Their complexion remained unchanged and there were no skin lesions. This represented a failure in their quest for a clear complexion, leading to feelings of disappointment, despair, and even depression.

Most of the subjects with low self-esteem were single, and the discomfort caused by the adverse cutaneous effects of depigmentation diminished their own perception of beauty and their seductive impulse, especially compared to models from the Western beauty canon. This leads to a feeling of failure, frustration, lack of self-confidence, an inferiority complex, and therefore a drop in self-esteem in these subjects, who are nonetheless in search of partners [10–13, 29].

Dyschromia, stretch marks, and acne are unruly and conspicuous. They prevent victims from displaying their relational and seductive assets. Dyschromia, more specifically hyperpigmentation of the interphalangeal and ankle regions, is a flagrant sign of depigmentation failure. People with these lesions are mocked and ridiculed by society. All of this would further impair self-image and therefore self-esteem. These results are in line with those found by Njimegne et al. in a similar study in Yaoundé in 2019 [30]. This reflects the close link between perception of one's own beauty, well-being, and self-esteem.

## 5. Conclusion

Artificial skin depigmentation in the city of Cotonou generally affects young, female, single, civil union, or married subjects with secondary or higher education. They used several galenic forms of lightening products in combination and the majority had more than two skin lesions, essentially dyschromia, stretch marks, superficial mycosis, and acne. Skin lesions affected the quality of life of 93.7% of the participants. 11.2% had definite symptoms of anxiety, and 5.8% had definite symptoms of depression. 24.2% of the subjects had below-average self-esteem. Subjects who used various lightening products had more skin lesions, leading to an altered quality of life, the persistence of which led to psychiatric comorbidities.

## Figures and Tables

**Table 1:** Distribution of the quality of life score among 330 subjects undergoing artificial skin depigmentation in the city of Cotonou in 2020, according to sociodemographic data, the number and monthly cost of lightening cosmetics used, and the number of skin complications.

	Quality of life impairment (%)					*P* value
	None	Weak	Moderate	Important	Extremely important	
	*n* = 21	*n* = 92	*n* = 126	*n* = 67	*n* = 24	
*Age (years)*						0.0001
15–20	28.6	13	10.3	4.5	8.3	
20–25	0	6.5	13.5	**22.4**	**37.5**	
25–30	19	28.2	17.4	**23.9**	8.3	
30–35	14.3	16.3	15.1	7.5	8.3	
35–40	19	10.9	16.7	9	16.7	
40–45	14.3	12	6.3	10.4	0	
45–50	4.8	9.8	4.8	14.9	4.2	
50 and more	0	3.3	15.9	7.4	16.7	
Total	100	100	100	100	100	
*Gender*						0.002
Female	38.1	64.1	73.8	**77.6**	**83.3**	
Male	61.9	35.9	26.2	22.4	16.7	
Total	100	100	100	100	100	
*Marital status*						0.016
Single	47.6	41.3	35.7	**49.3**	**62.5**	
Married	23.8	29.3	21.4	31.3	16.7	
Divorced	9.5	0	13.5	7.5	8.3	
Widow	0	4.4	7.1	1.5	4.2	
Civil partnership	19.1	25	22.3	10.4	8.3	
Total	100	100	100	100	100	
*Level of education*						<0.001
Unschooled	19	5.4	1.6	0	0	
Literate	0	15.2	11.9	1.5	0	
Primary	0	10.9	15	1.5	0	
Secondary	28.6	51.1	41.3	**34.3**	**20.8**	
High	52.4	17.4	30.2	**62.7**	**79.2**	
Total	100	100	100	100	100	
*Number of lightening products*						<0.001
One	23.8	13	3.2	0	0	
Two	**42.9**	**62**	**42.9**	22.4	16.7	
Three	19	18.5	**42.9**	**49.2**	**33.3**	
More than three	14.3	6.5	11	**28.4**	**50**	
Total	100	100	100	100	100	
*Number of skin complications*						<0.001
None	**71.4**	0	0.8	0	0	
One	19	**27.2**	5.6	4.5	0	
Two	9.6	**28.2**	**35.7**	**40.3**	16.7	
More than two	0	**44.6**	**57.9**	**55.2**	**83.3**	
Total	100	100	100	100	100	
Monthly cost of lightening products (FCFA)						<0.001
0–5000	23.8	**28.3**	20.6	1.5	0	
5000–10000	33.3	**48.9**	**38.1**	28.4	20.8	
10000–20000	33.3	20.6	**31.7**	**58.2**	**45.8**	
20000 and more	9.6	2.2	9.6	11.9	**33.4**	
Total	100	100	100	100	100	

The values in bold represent the proportions of variables that are significantly associated with quality life impairment.

**Table 2:** Distribution of the anxiety score among 330 subjects undergoing artificial skin depigmentation in the city of Cotonou in 2020 according to sociodemographic data, the number and monthly cost of lightening cosmetics used, and the number of skin complications.

	Anxiety (%)			*P* value
	Absent	Doubtful	Certain	
	*n* = 99	*n* = 194	*n* = 37	
Age (years)				<0.001
15–20	19.2	7.2	8.1	
20–25	15.1	14.9	8.1	
25–30	30.3	20.1	2.8	
30–35	9.1	16	10.8	
35–40	10.1	15,5	13.5	
40–45	7.1	10.3	5.4	
45–50	8.1	6.7	**16.2**	
50 and more	1	9.3	**35.1**	
Total	100	100	100	
*Gender*				0.046
Female	64.6	70.1	**86.5**	
Male	35.4	29.9	13.5	
Total	100	100	100	
*Marital status*				<0.001
Single	53.5	39.7	**29.7**	
Married	27.3	24.2	**27**	
Divorced	1	9.3	18.9	
Widow	1	3.6	18.9	
Civil partnership	17.2	23.2	5.5	
Total	100	100	100	
*Level of education*				0.009
Unschooled	5	3.1	0	
Literate	16.2	6.7	2.7	
Primary	7.1	10.8	5.4	
Secondary	45.4	35.6	**51.4**	
High	26.3	43.8	**40.5**	
Total	100	100	100	
*Occupation*				<0.001
Wage earners	17.2	2.7	**32.4**	
Entrepreneur	8.1	11.3	18.9	
Retired	0	1.1	18.9	
Shopkeepers	15.1	20.1	**21.6**	
Craftsman	23.2	14.9	0	
Housewife	11.1	9.3	0	
Secondary education student	17.2	3.6	0	
High education student	8.1	16	8.2	
Total	100	100	100	
*Number of lightening products*				<0.001
One	14.1	3.6	0	
Two	52.5	40.7	21.7	
Three	22.2	42.8	29.7	
More than three	11.2	12.9	**48.6**	
Total	100	100	100	
*Number of skin complications*				<0.001
None	5	5.7	0	
One	16.2	10.8	5.4	
Two	48.5	25.8	16.2	
More than two	30.3	57.7	**78.4**	
Total	100	100	100	
*Monthly cost of lightening products (FCFA)*				<0.001
0–5000	35.4	10.8	5.4	
5000–10000	45.5	39.2	8.1	
10000–20000	10	42.8	**62.2**	
20000 and more	9.1	7.2	**24.3**	
Total	100	100	100	

The bold values represent the proportions of variables that are significantly associated with anxiety.

**Table 3:** Distribution of the depression score among 330 subjects undergoing artificial skin depigmentation in the city of Cotonou in 2020 according to sociodemographic data, the number and monthly cost of lightening cosmetics used, and the number of skin complications.

	Depression (%)			*P* value
	Absent	Doubtful	Certain	
	*n* = 160	*n* = 151	*n* = 19	
Age (years)				0.03
15–20	17.5	5.4	0	
20–25	11.2	16.6	**21.1**	
25–30	23.8	19.9	10.5	
30–35	13.8	13.2	10.5	
35–40	10.6	15.2	**26.3**	
40–45	10	7.9	5.3	
45–50	9.3	6.6	10.5	
50 and more	3.8	15.2	15.8	
Total	100	100	100	
*Gender*				0.179
Female	65.6	74.2	78.9	
Male	34.4	25.8	21.1	
Total	100	100	100	
*Marital status*				0.006
Single	46.9	39.7	**31.6**	
Married	27.5	23.8	**21**	
Divorced	3	11.3	**21.1**	
Widow	1.3	7.3	10.5	
Civil partnership	21.3	17.9	15.8	
Total	100	100	100	
*Level of education*				0.03
Unschooled	5.6	0.7	5.3	
Literate	10.6	8.6	0	
Primary	8.8	9.2	10.5	
Secondary	45.6	35.8	**31.6**	
High	29.4	45.7	**52.6**	
Total	100	100	100	
*Occupation*				<0.001
Wage earners	17.5	24.5	**52.6**	
Entrepreneur	8.1	14.6	10.5	
Retired	0.6	5.3	0	
Shopkeepers	20	15.9	**31.6**	
Craftsman	21.3	11.9	0	
Housewife	10	8.6	0	
Secondary education student	13.1	2	0	
Higher education student	9.4	17.2	5.3	
Total	100	100	100	
*Number of lightening products*				<0.001
One	8.1	4.6	5.3	
Two	55	32.5	10.5	
Three	27.5	**43.7**	**31.6**	
More than three	9.4	19.2	**52.6**	
Total	100	100	100	
*Number of skin complications*				<0.001
None	4.4	4.6	10.5	
One	22.5	2	0	
Two	32.5	31.8	21.1	
More than two	40.6	61.6	**68.4**	
Total	100	100	100	
*Monthly cost of lightening products (FCFA)*				<0.001
0–5000	24.4	12.6	0	
5000–10000	46.2	31.8	10.5	
10000–20000	21.9	45.7	**63.2**	
20000 and more	7.5	9.9	**26.3**	
Total	100	100	100	

The bold values represent the proportions of the variables that are significantly associated with depression.

**Table 4:** Distribution of the self-esteem score among 330 subjects undergoing artificial skin depigmentation in the city of Cotonou in 2020 according to sociodemographic data, the number and monthly cost of lightening cosmetics used, and the number of skin complications.

	Self-esteem impairment (%)					*P* value
	Very weak	Weak	Medium	Important	Very important	
	*n* = 3	*n* = 77	*n* = 177	*n* = 72	*n* = 1	
Age (years)						0.27
15–20	66.7	10.4	13	6.9	0	
20–25	0	19.5	13.6	8.3	0	
25–30	33.3	18.2	20.3	26.4	100	
30–35	0	9	16.4	9.7	0	
35–40	0	11.7	14.1	15.3	0	
40–45	0	10.4	7.9	9.7	0	
45–50	0	9.1	9	5.6	0	
50 and more	0	11.7	5.7	18.1	0	
Total	100	100	100	100	100	
*Gender*						0.2
Female	100	77.9	69.5	62.5	100	
Male	0	22.1	30.5	37.5	0	
Total	100	100	100	100	100	
*Marital status*						0.006
Single	**66.7**	**41.6**	44.6	38.9	0	
Married	0	22	26.5	27.8	0	
Divorced	0	7.8	4	18	0	
Widow	0	11.7	3.4	0	0	
Civil partnership	33.3	16.9	21.5	15.3	100	
Total	100	100	100	100	100	
*Level of education*						<0.001
Unschooled	0	9.1	1.7	1.4	0	
Literate	0	20.8	6.8	2.8	0	
Primary	0	15.6	10.1	0	0	
Secondary	**100**	**32.5**	46.9	30.5	0	
High	0	22	34.5	65.3	100	
Total	100	100	100	100	100	
*Occupation*						<0.001
Wage earners	0	7.8	19.8	45.8	100	
Entrepreneur	0	7.8	11.3	15.3	0	
Retired	0	9.1	1.1	0	0	
Shopkeepers	**66.7**	**23.3**	19.2	11.1	0	
Craftsman	**33.3**	**15.6**	19.2	6.9	0	
Housewife	0	**19.5**	6.8	2.9	0	
Secondary education student	0	6.5	7.9	6.9	0	
High education student	0	10.4	14.7	11.1	0	
Total	100	100	100	100	100	
*Number of lightening products*						0.024
One	0	7.8	6.2	4.2	100	
Two	33.3	**46.8**	43.5	34.7	0	
Three	0	**31.1**	33.9	44.4	0	
More than three	**66.7**	14.3	16.4	16.7	0	
Total	100	100	100	100	100	
*Number of skin complications*						0.021
None	0	3.9	5.6	4.2	0	
One	0	7.8	11.3	16.7	100	
Two	0	23.4	31.1	43	0	
More than two	**100**	**64.9**	52	36.1	0	
Total	100	100	100	100	100	
*Monthly cost of lightening products (FCFA)*						0.025
0–5000	33.3	28.6	15.8	8.3	100	
5000–10000	0	**32.4**	42.4	33.3	0	
10000–20000	**66.7**	**29.9**	33.9	43.1	0	
20000 and more	0	9.1	7.9	15.3	0	
Total	100	100	100	100	100	

The bold values represent the proportions of variables that are significantly associated with self esteem impairment.

## Data Availability

The data used to support the findings of this study are available from the corresponding author upon request.
